# Supplementary material for: Cryptic susceptibility to penicillin/β-lactamase inhibitor combinations in emerging multidrug-resistant, hospital-adapted Staphylococcus epidermidis lineages
Source: Nat Commun. 2023 Oct 14;14:6479. doi: 10.1038/s41467-023-42245-y (PMC10576800; doi:10.1038/s41467-023-42245-y)
Supplement: Supplementary file 1 — Supplementary Information [file 41467_2023_42245_MOESM1_ESM.pdf]

Supplementary Table 1 | Alterations in the β-lactamase enzyme and native PBPs in methicillin-resistant *Staphylococcus epidermidis* ST2-mixed, ST5, and ST23 isolates that were phenotypically resistant, but genotypically predicted to be susceptible

**Study name**	**Country**	**Location**	**Isolation year**	**MLST**	**Hospital clone**	**Genotype**	**AMX MIC (µg ml-1) + 15 µg ml-1 CLA**	***blaZ* (no. copies)**	**PBP1**	**PBP2**	**PBP3**	**SERP_RS06395**
BPH0724	Australia	VIC-A	2009	2	ST2-mixed	S2	0.125	1	Ref.	Ref.	Ref.	Ref.
AUS16	Australia	VIC-E	2017	2	ST2-mixed	S2	8	1	-	-	Q145K	-
GER21	Germany	GER-T	2017	5	ST5/ST87	S2	0.5	1	Ref.	Ref.	Ref.	Ref.
BPH0723	Australia	VIC-A	2009	5	ST5/ST87	S2	8	1	-	-	-	-
AUS27	Australia	VIC-E	2017	23	ST23	S2	4	1	Ref.	Ref.	Ref.	Ref.
DEN22	Denmark	DEN-G	2016	23	ST23	S2	16	0[a]	-	S699L	-	-
DEN30	Denmark	DEN-H	2017	23	ST23	S2	32	0[a]	-	S699L	-	-
DEN31	Denmark	DEN-H	2017	23	ST23	S2	32	0[a]	-	S699L	-	-
DEN35	Denmark	DEN-H	2017	23	ST23	S2	32	0[a]	-	S699L	-	-
GER08	Germany	GER-H	2015	23	ST23	S2	32	0[a]	D718V	S699L	-	-

[a]DEN24, an unrelated S2 isolate belonging to ST23, also lacked *blaZ* but was phenotypically susceptible to amoxicillin/clavulanic acid.

Supplementary Table 2 | Short-read sequence data and annotated draft genomes

Study name	Study accession	Sample accession	Experiment accession	Run accession	Genome accession
BPH0719	PRJNA898869	SAMN32886138	-[a]	-[a]	JAQOSM000000000
BPH0719-Founder	PRJNA898869	SAMN33391325	SRX19450403	SRR23563466	JARBFR000000000
BPH0719-E1-D01	PRJNA898869	SAMN31634170	SRX18190545	SRR22212603	JAPFLX000000000
BPH0719-E1-D03	PRJNA898869	SAMN31634171	SRX18190546	SRR22212602	JAPFLW000000000
BPH0719-E1-D11	PRJNA898869	SAMN31634172	SRX18190552	SRR22212596	JAPFLV000000000
BPH0719-E2-D01	PRJNA898869	SAMN31634173	SRX18190553	SRR22212595	JAPFLU000000000
BPH0719-E2-D03	PRJNA898869	SAMN31634174	SRX18190554	SRR22212594	JAPFLT000000000
BPH0719-E2-D22	PRJNA898869	SAMN31634175	SRX18190555	SRR22212593	JAPFLS000000000
BPH0719-E3-D01	PRJNA898869	SAMN31634176	SRX18190556	SRR22212592	JAPFLR000000000
BPH0719-E3-D03	PRJNA898869	SAMN31634177	SRX18190557	SRR22212591	JAPFLQ000000000
BPH0719-E3-D27	PRJNA898869	SAMN31634178	SRX18190558	SRR22212590	JAPFLP000000000
DEN09	PRJNA898869	SAMN32886139	-[a]	-[a]	JAQOSN000000000
DEN09-Founder	PRJNA898869	SAMN33391326	SRX19450404	SRR23563465	JARBFS000000000
DEN09-E1-D01	PRJNA898869	SAMN31634179	SRX18190559	SRR22212589	JAPFLO000000000
DEN09-E1-D25	PRJNA898869	SAMN31634180	SRX18190547	SRR22212601	JAPFLN000000000
DEN09-E2-D01	PRJNA898869	SAMN31634181	SRX18190548	SRR22212600	JAPFLM000000000
DEN09-E2-D30	PRJNA898869	SAMN31634182	SRX18190549	SRR22212599	JAPFLL000000000
DEN09-E3-D01	PRJNA898869	SAMN31634183	SRX18190550	SRR22212598	JAPFLK000000000
DEN09-E3-D29	PRJNA898869	SAMN31634184	SRX18190551	SRR22212597	JAPFLJ000000000

[a]Illumina paired-end reads of BPH0719 and DEN09 were retrieved from Lee et al. (BioProjects PRJEB12090 and PRJNA470752)[4].
