# Supplementary material for: Cryptic susceptibility to penicillin/β-lactamase inhibitor combinations in emerging multidrug-resistant, hospital-adapted Staphylococcus epidermidis lineages
Source: Nat Commun. 2023 Oct 14;14:6479. doi: 10.1038/s41467-023-42245-y (PMC10576800; doi:10.1038/s41467-023-42245-y)
Supplement: Supplementary file 3 — Description of Additional Supplementary Files [file 41467_2023_42245_MOESM3_ESM.pdf]

Description of Additional Supplementary Files

Supplementary Data 1: This file contains information about the 227 *S. epidermidis* isolates used in this study.

Supplementary Data 2: This file contains additional information about the 138 MRSE BPH0662 clone, ST2-mixed, ST5, and ST23 isolates from Australia, Denmark, and Germany.

Supplementary Data 3: This file contains the PhyML tree file for 138 MRSE BPH0662 clone, ST2-mixed, ST5, and ST23 isolates from Australia, Denmark, and Germany in Newick format.
