# Supplementary material for: Cryptic susceptibility to penicillin/β-lactamase inhibitor combinations in emerging multidrug-resistant, hospital-adapted Staphylococcus epidermidis lineages
Source: Nat Commun. 2023 Oct 14;14:6479. doi: 10.1038/s41467-023-42245-y (PMC10576800; doi:10.1038/s41467-023-42245-y)
Supplement: Supplementary file 7 — Reporting Summary [file 41467_2023_42245_MOESM7_ESM.pdf]

nature portfolio

Corresponding author(s): Jesper Larsen

Last updated by author(s): Sep 15, 2023

Reporting Summary

Nature Portfolio wishes to improve the reproducibility of the work that we publish. This form provides structure for consistency and transparency in reporting. For further information on Nature Portfolio policies, see our Editorial Policies and the Editorial Policy Checklist.

Statistics

For all statistical analyses, confirm that the following items are present in the figure legend, table legend, main text, or Methods section.

n/a	Confirmed	
☐	☒	The exact sample size (*n*) for each experimental group/condition, given as a discrete number and unit of measurement
☐	☒	A statement on whether measurements were taken from distinct samples or whether the same sample was measured repeatedly
☐	☒	The statistical test(s) used AND whether they are one- or two-sided<br>*Only common tests should be described solely by name; describe more complex techniques in the Methods section.*
☒	☐	A description of all covariates tested
☒	☐	A description of any assumptions or corrections, such as tests of normality and adjustment for multiple comparisons
☐	☒	A full description of the statistical parameters including central tendency (e.g. means) or other basic estimates (e.g. regression coefficient) AND variation (e.g. standard deviation) or associated estimates of uncertainty (e.g. confidence intervals)
☐	☒	For null hypothesis testing, the test statistic (e.g. *F*, *t*, *r*) with confidence intervals, effect sizes, degrees of freedom and *P* value noted<br>*Give P values as exact values whenever suitable.*
☒	☐	For Bayesian analysis, information on the choice of priors and Markov chain Monte Carlo settings
☒	☐	For hierarchical and complex designs, identification of the appropriate level for tests and full reporting of outcomes
☒	☐	Estimates of effect sizes (e.g. Cohen's *d*, Pearson's *r*), indicating how they were calculated

Our web collection on statistics for biologists contains articles on many of the points above.

Software and code

Policy information about availability of computer code

Data collection	No software was used to collect data in our study.
Data analysis	SPAdes v3.15<br>BLASTN<br>MUSCLE<br>NASP v1.0 (including Burrows-Wheeler Alignment tool,GATK Unified Genotyper, and NUCmer)<br>PHASTER<br>Gubbins v2.3.4<br>PhyML v3.0 (including GTR model of nucleotide substitution and aBayes)<br>The custom R code for empirical ECOFF determination is available through Zenodo (https://doi.org/10.5281/zenodo.8344500)<br>NCBI Prokaryotic Genome Annotation Pipeline<br>NCBI Conserved Domain database v3.20 (including RPS-BLAST)<br>GraphPad Prism v8.3

For manuscripts utilizing custom algorithms or software that are central to the research but not yet described in published literature, software must be made available to editors and reviewers. We strongly encourage code deposition in a community repository (e.g. GitHub). See the Nature Portfolio guidelines for submitting code & software for further information.

Data

Policy information about availability of data

All manuscripts must include a data availability statement. This statement should provide the following information, where applicable:

- Accession codes, unique identifiers, or web links for publicly available datasets
- A description of any restrictions on data availability
- For clinical datasets or third party data, please ensure that the statement adheres to our policy

S. epidermidis short-read sequence data and annotated genomes generated in this study have been deposited in the European Nucleotide Archive/NCBI Sequence Read Archive under BioProject PRJNA898869 and the accession numbers are provided in Supplementary Table 2. Sequence data from other sources comprised the closed genomes and plasmids of BPH0662 (GenBank accession no. NZ_LT571449.1, NZ_LT614820.1, NZ_LT571450.1, NZ_LT571451.1, and NZ_LR736240.1), PM221 (GenBank accession no. NZ_HG813242.1, NZ_HG813246.1, NZ_HG813245.1, NZ_HG813244.1, NZ_HG813243.1, and SEI (GenBank accession no. NZ_CP009046.1 and NZ_CP009047.1), Illumina paired-end reads of the remaining 224 S. epidermidis isolates (BioProjects PRJEB12090, PRJNA470534, and PRJNA470752), the mecA promoter and PBP2a-encoding gene in S. aureus strain COL (corresponding to nucleotide positions 39,643-41,718 in GenBank accession no. NC_002951), blaZ in S. aureus strain PC1 (corresponding to nucleotide positions 123-968 in GenBank accession no. M25252), and native PBP-encoding genes in S. epidermidis strain RP62A (pbp1, pbp2, pbp3, and SERP_RS06395 corresponding to nucleotide positions 741901-744228, 741901-744228, 1155781-1157871, and 1340351-1341256, respectively, in GenBank accession no. NC_002976). Information about the 227 S. epidermidis isolates used in this study is provided in Supplementary Data 1. Additional information about the 138 MRSE BPH0662 clone, ST2-mixed, ST5, and ST23 isolates from Australia, Denmark, and Germany is provided in Supplementary Data 2. The PhyML tree file for 138 MRSE BPH0662 clone, ST2-mixed, ST5, and ST23 isolates from Australia, Denmark, and Germany is provided in Newick format in Supplementary Data 3.. Source data for Figs. 1-3 and Figs. 5-7 are provided with this paper.

Research involving human participants, their data, or biological material

Policy information about studies with human participants or human data. See also policy information about sex, gender (identity/presentation), and sexual orientation and race, ethnicity and racism.

Reporting on sex and gender	Information on sex and gender was not collected in our study.
Reporting on race, ethnicity, or other socially relevant groupings	Information on race, ethnicity, or other socially relevant groupings was not collected in our study.
Population characteristics	Not applicable (see above).
Recruitment	Not applicable (see above).
Ethics oversight	Not applicable (see above).

Note that full information on the approval of the study protocol must also be provided in the manuscript.

Field-specific reporting

Please select the one below that is the best fit for your research. If you are not sure, read the appropriate sections before making your selection.

☒ Life sciences ☐ Behavioural & social sciences ☐ Ecological, evolutionary & environmental sciences

For a reference copy of the document with all sections, see nature.com/documents/nr-reporting-summary-flat.pdf

Life sciences study design

All studies must disclose on these points even when the disclosure is negative.

Sample size	We used a previously described global collection of 227 S. epidermidis isolates from 96 hospitals in 24 countries (Reference #4). We did not perform sample size calculations as the total number of isolates was determined by what was available in the collection. For animal experiments, sample sizes were based on previous experience with the mouse infection model (Reference #6) as well as on the principles of the 3Rs (Replacement, Reduction, and Refinement), of which Reduction refers to any strategy that will result in the lowest possible number of animals being used to answer the research question. All conclusions were supported by statistical analyses.
Data exclusions	No data were excluded from our analyses.
Replication	In general, experiments were replicated two to six times in accord with the generally accepted standards in the field. All attempts at replication were successful.
Randomization	Animals were randomly allocated into groups. Randomization was not relevant for the other experiments as they did not involve allocation to different groups.
Blinding	For animal experiments, blinding was not relevant as bacterial counts are quantitative and therefore not subject to human bias. Blinding was not relevant for the other experiments as they did not involve allocation to different groups.

Reporting for specific materials, systems and methods

We require information from authors about some types of materials, experimental systems and methods used in many studies. Here, indicate whether each material, system or method listed is relevant to your study. If you are not sure if a list item applies to your research, read the appropriate section before selecting a response.

Materials & experimental systems

n/a	Involved in the study
☒	☐ Antibodies
☒	☐ Eukaryotic cell lines
☒	☐ Palaeontology and archaeology
☐	☒ Animals and other organisms
☒	☐ Clinical data
☒	☐ Dual use research of concern
☒	☐ Plants

Methods

n/a	Involved in the study
☒	☐ ChIP-seq
☒	☐ Flow cytometry
☒	☐ MRI-based neuroimaging

Animals and other research organisms

Policy information about studies involving animals; ARRIVE guidelines recommended for reporting animal research, and Sex and Gender in Research

Laboratory animals	Animal experiments were performed in the animal facility at Statens Serum Institut using 6-8 weeks old female NMRI mice (Envigo). Mice were housed individually in ventilated cages at a constant temperature (22±2°C) and relative humidity (55±10%), with a 12:12 h light:dark cycle (light on 06.00-18.00h). Food and water were available ad libitum.
Wild animals	The study did not involve wild animals.
Reporting on sex	Information on sex was not relevant in our study.
Field-collected samples	The study did not involve field-collected samples.
Ethics oversight	Animal experiments were approved by the Danish Animal Experiments Inspectorate (2016-15-0201-01049).

Note that full information on the approval of the study protocol must also be provided in the manuscript.
